# Maternal Exposure to Endocrine-Disrupting Chemicals: Analysis of Their Impact on Infant Gut Microbiota Composition

**DOI:** 10.3390/biomedicines12010234

**Published:** 2024-01-19

**Authors:** Mirco Vacca, Francesco Maria Calabrese, Federica Loperfido, Beatrice Maccarini, Rosa Maria Cerbo, Eduardo Sommella, Emanuela Salviati, Luana Voto, Maria De Angelis, Gabriele Ceccarelli, Ilaria Di Napoli, Benedetta Raspini, Debora Porri, Elisa Civardi, Francesca Garofoli, Pietro Campiglia, Hellas Cena, Rachele De Giuseppe

**Affiliations:** 1 Department of Soil, Plant and Food Science, University of Bari Aldo Moro, 70126 Bari, Italy; mirco.vacca@uniba.it (M.V.); francesco.calabrese@uniba.it (F.M.C.); maria.deangelis@uniba.it (M.D.A.); 2 Laboratory of Dietetics and Clinical Nutrition, Department of Public Health, Experimental and Forensic Medicine, University of Pavia, 27100 Pavia, Italy; beatrice.maccarini01@universitadipavia.it (B.M.); lvoto4@gmail.com (L.V.); ilaria.dinapoli@unipv.it (I.D.N.); benedetta.raspini.nutrizione@gmail.com (B.R.); debporri@gmail.com (D.P.); hellas.cena@unipv.it (H.C.); rachele.degiuseppe@unipv.it (R.D.G.); 3 Neonatal Unit and Neonatal Intensive Care Unit, Fondazione IRCCS Policlinico San Matteo, 27100 Pavia, Italy; rm.cerbo@smatteo.pv.it (R.M.C.); e.civardi@smatteo.pv.it (E.C.); fragarofoli@gmail.com (F.G.); 4 Department of Pharmacy, University of Salerno, 84084 Fisciano, Italy; esommella@unisa.it (E.S.); esalviati@unisa.it (E.S.); pcampiglia@unisa.it (P.C.); 5 Human Anatomy Unit, Department of Public Health, Experimental and Forensic Medicine, University of Pavia, 27100 Pavia, Italy; gabriele.ceccarelli@unipv.it; 6 Clinical Nutrition Unit, General Medicine, Istituti Clinici Scientifici Maugeri IRCCS, 27100 Pavia, Italy

**Keywords:** endocrine disrupting chemicals, breastfeeding, gut microbiota, first 1000 days, bisphenol a, phthalates

## Abstract

Endocrine disruptors (EDCs) are chemicals that interfere with the endocrine system. EDC exposure may contribute to the development of obesity, type 2 diabetes, and cardiovascular diseases by impacting the composition of an infant’s gut microbiota during the first 1000 days of life. To explore the relationship between maternal urinary levels of Bisphenol-A and phthalates (UHPLC-MS/MS), and the composition of the infant gut microbiota (16S rDNA) at age 12 months (T_3_) and, retrospectively, at birth (T_0_), 1 month (T_1_), and 6 months (T_2_), stool samples from 20 infants breastfed at least once a day were analyzed. Metataxonomic bacteria relative abundances were correlated with EDC values. Based on median Bisphenol-A levels, infants were assigned to the over-exposed group (O, n = 8) and the low-exposed group (B, n = 12). The B-group exhibited higher gut colonization of the *Ruminococcus torques* group genus and the O-group showed higher abundances of *Erysipelatoclostridium* and *Bifidobacterium breve*. Additionally, infants were stratified as high-risk (HR, n = 12) or low-risk (LR, n = 8) exposure to phthalates, based on the presence of at least three phthalates with concentrations exceeding the cohort median values; no differences were observed in gut microbiota composition. A retrospective analysis of gut microbiota (T_0_–T_2_) revealed a disparity in β-diversity between the O-group and the B-group. Considering T_0_–T_3_, the Linear Discriminant Effect Size indicated differences in certain microbes between the O-group vs. the B-group and the HR-group vs. the LR-group. Our findings support the potential role of microbial communities as biomarkers for high EDC exposure levels. Nevertheless, further investigations are required to deeply investigate this issue.

## 1. Introduction

Endocrine Disruptors (EDCs), which encompass Bisphenol A (BPA) and phthalates, represent a class of chemicals that are capable of interfering with the normal functioning of the endocrine system. This system plays a pivotal role in regulating various bodily processes, including growth, development, metabolism, and reproduction [1,2]. Consequently, EDCs have the potential to influence the key components of human metabolism, affecting critical organs such as the hypothalamus, adipose tissue, pancreatic beta cells, liver, and skeletal muscles [1,3]. During vital developmental stages, EDC exposure can induce epigenetic modifications at the germinal level, which may be passed down through generations [4]. Emerging evidence suggests that exposure to EDCs may contribute to the onset of obesity, metabolic syndrome, diabetes mellitus, and non-alcoholic fatty liver disease [1,5]. For instance, BPA and certain phthalates can mimic or interfere with the signaling pathways of hormones such as estrogens, androgens, glucocorticoids, thyroid hormones, and insulin, leading to tissue-specific effects, especially within adipose tissue, reproductive organs, thyroid, liver, and pancreas [2,6]. EDCs are prevalent in various consumer products, including plastics, food packaging, and personal care items, entering the body through ingestion, inhalation, and dermal exposure [1,7]. Due to differences in physiology, anatomy, and toxicokinetics between adults and children, fetuses and infants are more vulnerable to EDC exposure [8,9] and are at a higher risk of developing the above listed pathologies. This heightened susceptibility begins during pregnancy, where EDC exposure can affect the child’s genetics through epigenetic modifications (DNA methylation, histone modifications, and non-coding RNA-mediated regulation), potentially impacting the child’s long-term health [4,8,10,11,12]. Prenatal and postnatal exposure to EDCs becomes even more critical during “the first 1000 days” birth conception, as this period represents the highest vulnerability phase in an individual’s life [13]. Events occurring during these early stages significantly shape the development of chronic diseases throughout one’s life, emphasizing the substantial impact of the maternal environment on the child’s future life [13].

One way in which EDCs may contribute to these diseases is by altering the composition of the gut microbiota, that is, the complex polymicrobial ecosystem colonizing the digestive tract [14]. The gut microbiota plays a pivotal role in regulating metabolism and energy balance by extracting nutrients from food and producing metabolites that can influence the host’s metabolic processes [15]. Research has discussed that exposure to EDCs can disrupt the gut microbiota’s composition, leading to changes in energy metabolism and weight gain [16].

In the context of the first 1000 days, breast milk is one of the most important sources of EDC exposure for newborns, since these chemicals—metabolized and accumulating in the human body—have been detected in the mother’s milk, serum, hair, and placenta [17]. As a consequence, the entero-mammary circle is believed to facilitate the transfer of these chemicals, as certain EDCs can traverse the gut barrier, enter the bloodstream, and be transported to the mammary glands, subsequently appearing in breast milk [18]. Research has shown that phthalates, including di-(2-ethylhexyl)-phthalate (DEHP) diisononylphthalate (DINP), and bisphenol A (BPA) are among the most common EDCs detected in breast milk [19,20,21]. The EDC concentration in human milk can be influenced by various maternal exposure factors, such as lifestyle (e.g., dietary habits, smoking), place of residence, occupation, and the types of personal products used in daily life [22]. A recent cross-sectional study examined EDC levels and their major determinants of daily exposure (e.g., dietary choices, physical activity, smoking) in the A.MA.MI (*Alimentazione MAmma e bambino nei primi Mille giorni*) cohort, assessing urinary BPA and phthalate levels in 45 women [2]. The study revealed that most women had been exposed to common sources of EDCs, with 63.4% using plastic personal care products, 53.7% encountering polyvinyl chloride in their living or work environments, and 17.8% being exposed to synthetic materials during recreational activities [2]. Additionally, the consumption of packaged food in plastic containers or wrapped in plastic film, as well as canned or tetra-packed food, was common among all women [2]. Consequently, a positive and significant association was observed between the consumption of sauces and dressings in plastic containers and monoethyl-phthalate exposure, while the association between BPA levels and takeaway or cooked food consumption neared statistical significance [2].

Considering breast milk as a primary vehicle of EDCs from mother to infant, this study aims to investigate maternal exposure levels to phthalates and BPA and to assess the association between maternal EDC exposure and the composition of the infant’s intestinal microbiota at 1 year of age, as well as at birth, 1 month, and 6 months of age, retrospectively. Based on our study purpose, in the A.MA.MI. cohort, we identified infants who had been breastfed at least once a day up to 12 months of age and related samples were compared considering a different EDC exposure risk.

## 2. Materials and Methods

### 2.1. Study Design

The present study is part of the longitudinal, prospective study, named A.MA.MI (*Alimentazione MAmma e bambino nei primi MIlle giorni*; ClinicalTrials.gov identifier: NCT04122612), investigating the correlation between the infant gut microbiota composition and maternal/infant lifestyle and environmental factors, including the urinary maternal levels of EDCs, from conception to the first year of life, at different follow-up stages (T_0_, at delivery; T_1_, 1 month after delivery; T_2_, 6 months after the delivery; T_3_, 12 months after the delivery) (Figure S1) [13]. The study was approved by the Human Ethics Committee of *Fondazione IRCCS Policlinico S. Matteo of Pavia* (protocol number: 20180022618; 12 June 2018; addendum, protocol number: 20190068597, 1 August 2019). The research was conducted in a group of 63 mother–infant pairs referred to the Neonatal Unit of the *Fondazione IRCCS Policlinico San Matteo*, Pavia (Italy) from birth to 1 year of age, according to the Good Clinical Practice guidelines. Written informed consent of the parent/legal guardian was provided. The complete study design and the study protocol were already previously detailed by Raspini and colleagues [13].

Herein, 45 mother–infant pairs (28% of dropouts) were considered 12 months after delivery, corresponding to the 4th sampling time (T_3_) of the A.MA.MI project and according to the following inclusion criteria: infants of both sexes, born by natural or caesarean delivery, gestational age between 37 and 42 completed weeks, Italian-speaking parents, the ability of the parent/guardian to give informed consent, the ability of the mother to respond to the structured interview/questionnaires; and exclusion criteria: infants or mothers with genetic/congenital diseases, infants hospitalized in the neonatal intensive care unit immediately after birth; infants selected for other clinical studies, the presence of gestational diabetes, the presence of hyperthyroidism during pregnancy.

Infant fecal samples and maternal urinary samples were collected and subjected to the gut microbiota composition and EDC assessment, respectively. To reduce bias, the administration of antibiotics, probiotics, or prebiotics in the past 3 months before the fecal sample delivery was considered as further exclusion criteria.

In addition, we assessed maternal exposure to EDCs by analyzing the levels in single-spot urinary samples during T_3_. Specifically, we measured the concentrations of Bisphenol A (BPA), monomethyl phthalate (MEP) as a metabolite of diethyl phthalate (DEP), mono isobutyl-phthalate (MIbP) as a metabolite of n-butyl phthalate (DnBP), and metabolites of di(2-ethylhexyl) phthalate (DEHP), including mono (2-ethyl-5-hydroxylhexyl) phthalate (MEHHP) and mono (2-ethylhexyl) phthalate (MEHP). We also examined mono benzyl phthalate (MBzP) as a metabolite of benzyl butyl phthalate (BBP). Furthermore, we collected data on the (i) medical history and demographic characteristics of the mothers, including their occupation (housewife vs. other employment), level of education (tertiary education vs. non-tertiary education), and the presence of chronic disease (yes vs. no); (ii) anthropometric parameters; (iii) lifestyle habits, including food consumption frequency/eating habits, physical activity level, and smoking habits (Table S2); and (iv) dietary exposure to EDCs (Table S3).

Regarding infants, postnatal variables, such as type of feeding and timing of introduction of complementary foods, were also evaluated by questionnaire.

Based on the type of breastfeeding, infants who had been fed breast milk at least once a day up to 12 months of age were identified (n = 20). Only for these 20 children, the composition of the intestinal microbiota was taken into consideration and related to maternal EDC levels.

Infants were then divided into different groups, based on the maternal levels of urinary BPA and phthalates (MEP, MIbP, MEHHP, MBzP, and MEHP), which indicated maternal exposure to EDCs and were previously described by Di Napoli et al. in the A.MA.MI cohort [2]. In particular, (i) infants of mothers who reported urinary BPA levels greater than the median value of the A.MA.MI cohort (0.96 µg/g creatinine) (infants of mothers highly exposed to BPA, O group); (ii) infants of mothers who reported urinary BPA levels at or lower than the median value of the A.MA.MI cohort (0.96 µg/g creatinine) (infants of mothers with low exposure to BPA, B group); (iii) infants of mothers who had levels above the relative median value for at least 3 out of 5 phthalates (infants of mothers highly exposed to phthalates, HR group); (iv) infants of mothers who had levels at or below the relative median value for at least 3 out of 5 phthalates (infants of mothers with low exposure to phthalates, LR group). For phthalate levels, the median values were reported as follows: MEP: 7.73 µg/g creatinine; MIbP: 0.89 µg/g creatinine; MEHHP: 1.24 µg/g creatinine; MBzP: 0.64 µg/g creatinine (Table 1). The infant gut microbiota composition was compared between the different exposure classes.

### 2.2. Maternal Anthropometric Parameters

During T_3_, we assessed maternal anthropometric parameters, including height (cm), weight (kg), hip (cm), and waist circumference (WC, cm). These measurements were conducted following standardized procedures outlined elsewhere [2]. Subsequently, we computed the Body Mass Index (BMI), defined as the weight in kilograms divided by the square of the height in meters. Additionally, we calculated the Waist-to-Hip Ratio (WHR) and the Waist (cm) to Height (cm) Ratio (WHtR). These indices are recognized as effective predictors of abdominal adiposity in adults [2].

### 2.3. Maternal Phthalate, BPA, and Creatinine Level Determination

Bisphenol A (BPA) and various phthalate concentrations, including monomethyl phthalate (MEP), mono isobutyl-phthalate (MIbP), mono (2-ethyl-5-hydroxylhexyl) phthalate (MEHHP), mono benzyl phthalate (MBzP), and mono (2-ethylhexyl) phthalate (MEHP), were measured in clean, mid-stream urine samples, as per the method described by Lee et al. [23]. Concurrently, creatinine concentrations were determined. The urine samples were collected in polypropylene cups intended for urine culture, and participants were instructed to store the cups in a cool environment until they could be transported. These urine samples were then transported under refrigeration, divided into tubes made from phthalate-free materials, and stored at −80 °C until the time of analysis.

BPA and phthalate concentrations were quantified using RP-UHPLC-MS/MS (Shimadzu, Milan, Italy) on a Nexera UHPLC coupled online to a triple quadrupole LCMS8050 (Shimadzu, Kyoto, Japan). Detailed extraction and UHPLC-MS/MS conditions for the BPA and phthalate metabolites can be found elsewhere [2].

Creatinine levels were measured using routine methods on an Atellica^®^ CH Analyzer (Siemens Healthcare Diagnostics Inc., Tarrytown, NY, USA) and expressed as _mol/L. All urinary samples were considered acceptable because the creatinine concentrations were within the range of 0.3–3.0 g/L [24].

### 2.4. Type of Feeding and Dietary Habits of Infants

At T_3_, mothers/parents were interviewed by trained personnel to inquire about the type and duration of feeding (exclusive breastfeeding, non-exclusive breastfeeding, non-breastfeeding) and dietary habits, in terms of diversity and frequency of food groups consumed by the infant at 12 months of age, using the questionnaire developed by the International Fetal and Newborn Growth Consortium (INTERGROWTH) [25]. In the present research, only infants who had been fed breast milk at least once a day up to 12 months of age (n = 20) were considered.

### 2.5. Sample Processing for Gut Microbiota Analysis

The fecal samples collected were immediately stored at −80 °C in the Neonatal Intensive Care Unit at *Fondazione IRCCS Policlinico San Matteo* (Pavia, Italy) until processing started. Subsequently, the samples were shipped on dry ice to Genomix-4Life Srl, located at the Laboratory of Molecular and Genomic Medicine within the Campus of Medicine and Surgery (Baronissi, Salerno, Italy), where gene amplicon analysis was conducted. Total DNA was extracted from the stool samples using the Invimag Stool kit (Stratec Molecular GmbH, Berlin, Germany), following the manufacturer’s instructions. Sterilized distilled water was used as an extraction negative control. To ensure privacy, no clinical or personal information was included, except for the ID number assigned to each sample. The 16S rDNA gene amplification targeted the hypervariable V3-V4 regions of the 16S rDNA gene using the primers Forward 5′-CCT ACG GGNGGC WGC AG-3′ and Reverse 5′-GAC TAC HVGGG TAT CTA ATC C-3′. Detailed protocols for obtaining raw sequence files (fastq files) and filtering them based on quality control (FastQC) were previously outlined [26]. The 16S rDNA metabarcoding analysis was performed using QIIME2, and the SILVA 138 taxonomic database was used for bacterial taxonomy assignment.

### 2.6. Phylogenetic Investigation of Communities by Reconstruction of Unobserved States (PICRUSt2)

The PICRUSt analysis was conducted as previously detailed [27]. Briefly, for each sample, predictions of metabolic pathways were generated from the 16S rRNA marker gene data using the *‘Phylogenetic Investigation of Communities by Reconstruction of Unobserved States*’ (PICRUSt2) software 2.0 version, which was executed as a software extension within the QIIME2 library. The MetaCyc pathway abundances per sample were employed as input for a two-sided Welch test comparing the groups.

### 2.7. Statistical Analyses

Descriptive statistics were employed to summarize the data, presenting means and standard deviations (± SD) or medians with interquartile ranges (IQR) for quantitative variables, and relative frequencies for qualitative variables. Spearman’s rank correlation statistics were used to obtain non-parametric measures of association (R) between chemical concentrations and microbial relative abundances. To assess the β-diversity differences in gut microbiota composition between samples, a Permutational Multivariate Analysis of Variance (PERMANOVA) was conducted using the ‘adonis’ function from the ‘vegan’ package in the R environment. Microbiota features (Amplicon Sequence Variants, ASVs) associated with cohort stratification were evaluated using Linear Discriminant Analysis Effect Size (LEfSe) through the relevant function in the R environment. For the analysis of PICRUSt2 data, the ‘Volcano.Anal’ function was executed in the R environment, considering differences that were significant with a fold change (FC) greater than 2 folds and with a *p*-value (*p*) < 0.05. In all other statistical analyses, significance was determined by *p* < 0.05.

## 3. Results

### 3.1. Population

A population of 45 women enrolled in the A.MA.MI project and previously described [2] were taken into consideration at T_3_. In brief, 50% were under the age of 36 years and 12.9% had chronic conditions (e.g., rheumatoid arthritis, chronic migraine, hypothyroidism, or healthy carrier of beta-thalassemia). Regarding the ponderal status, 62.8% of the participants were within the normal weight range (reference interval: 18.5 kg/m^2^ ≤ BMI < 25 kg/m^2^), while 79.5% had a WC below 88 cm (cut-off value < 88.0 cm). Furthermore, all women showed a WHR at or less than 0.85 (cut-off value < 0.85); for 55.8% of them, the fat mass distribution index (WHtR) was at or less than 0.48 (cut-off value < 0.5) (Table S1). Finally, most of the mothers enrolled (87%) held occupations and spent 8 h a day at work, while only 12.9% of them were housewives. It is noteworthy that, for the present analysis, only women who breastfed their newborns at least once a day up to 12 months of age were identified (n = 20) and considered. 

### 3.2. Infants’ Gut Microbiota and Maternal Urinary EDC Correlations

**Figure 1:**
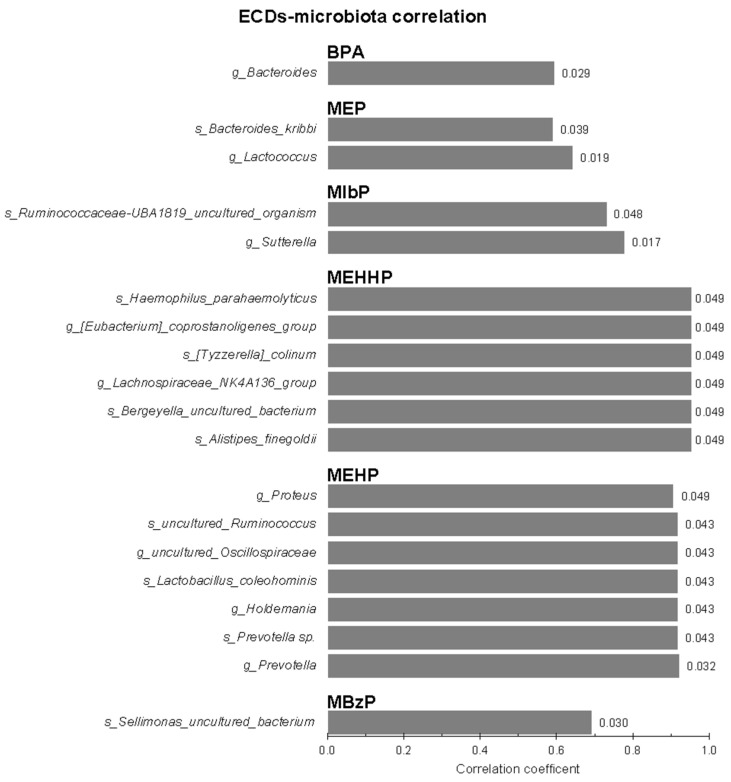
Based on Spearman’s rank correlation, significant (*p* < 0.05) correlation scores between the relative abundance of gut microbes found in stool samples of infants breastfed at least one a day up to 12 months of age and the EDC concentrations found in maternal urinary samples. Before the taxon name, the taxonomy level of the relative ASV (g, genus or s, species) is shown.

Stool samples of infants breastfed at least once a day up to 12 months of age were used for gut microbiota profiling and the obtained ASV-relative abundances were correlated with the EDC concentrations found in the relative maternal urines (Figure 1). By filtering for significance (*p* < 0.05), BPA positively correlated with the genus *Bacteroides*. MEP positively correlated with two different taxa, *Bacteroides kribbi* and the genus *Lactococcus*. MIbP positively correlated with uncultured species assigned to the *Ruminococcaceae*-UBA1819 genus and with the genus *Sutterella*. Both the two DEHP-deriving EDCs, i.e., MEHHP and MEHP, showed the highest number of positive correlations with various gut microbial taxa. In detail, MEHHP correlated with two different genera (*Eubacterium coprostanoligenes* group and the group NK4A136 of *Lachnospiraceae*) and four different species (*Haemophilus parahaemolyticus*, *Tyzzerella colinum*, *Bergeyella* sp., and *Alistipes finegoldii*), while MEHP correlated with four different genera (*Proteus*, uncultured *Oscillospiraceae*, *Holdemania*, and *Prevotella*) and two different species (uncultured *Ruminococcaceae* and *Prevotella* sp.). One positive correlation was found for MBzP with an uncultured species belonging to the *Sellimonas* genus.

### 3.3. Exposure to High BPA and EDC Concentrations via Breastfeeding

**Figure 2:**
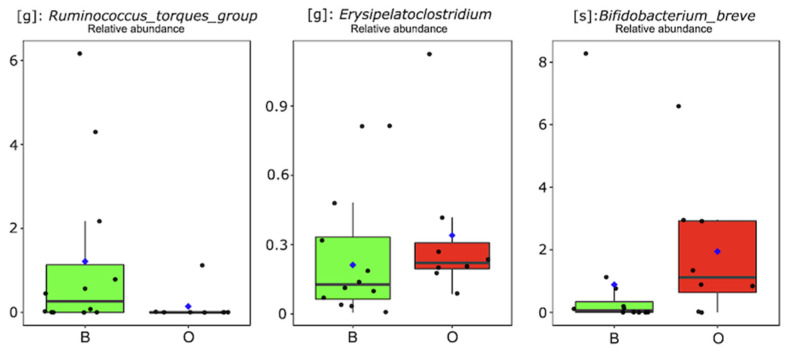
Gut microbiota differences between infants of mothers highly exposed to BPA (O group; BPA maternal levels > 0.96 µg/g creatinine) and infants of mothers less exposed to BPA (B group; BPA maternal levels ≤ 0.96 µg/g creatinine).

In the second step of the investigation, infants were stratified into two groups based on the BPA concentrations determined in their mothers’ urine samples. The label “high exposure to BPA” was assigned to infants whose mothers had a urinary BPA concentration higher than the cohort median value (Table 1). Infants were then allocated to the O/B group based on the detection of values above (O) or below (B) 0.96 mg/g creatinine. This approach resulted in eight infants in the O-group and 12 in the B-group. ASV-relative abundances were compared between O- and B-infants, revealing that only three different taxa were significantly different between the groups (Figure 2). Specifically, the genus *Ruminococcus torques* group was higher (*p* < 0.05) in B-infants, while *Erysipelatoclostridium* and *Bifidobacterium* breve showed a higher relative abundance in feces delivered by O-labeled infants. A similar approach was then applied to infants stratified for high-risk (HR) and low-risk (LR) exposure to a residual pattern of EDCs (i.e., MEP, MIbP, MEHHP, MEHP, and MBzP), based on maternal urine sample profiling. Mothers with at least three out of five EDCs co-present, with concentrations higher than the related median value (Table 1), determined HR labeling for their children. This resulted in eight infants being allocated to the HR group and 12 to the LR group; however, from the comparison between these groups, no significant differences (*p* > 0.05) were found in the gut microbiota of infants.

### 3.4. Retrospective Profiling of Infants’ Gut Microbiota

Maintaining the same subgrouping (O vs. B and HR vs. LR), differences in gut microbiota were retrospectively investigated. Therefore, in addition to fecal samples collected at 12 months of age (T_3_), the meconium (T_0_), and feces collected at 1 month (T_1_) and 6 months (T_2_) of infants’ age were also analyzed. Based on Jaccard’s index, β-diversity was examined for the comparison of the O-group against the B-group and the HR-group against the LR-group at all taxonomic levels (Table 2). Although specifically focused on the genus level, once again, the comparison of O against B revealed a significant difference in sample β-diversity (Table 2). Conversely, no difference was found when comparing HR β-diversity against LR for all taxonomic levels.

### 3.5. Potential Gut Microbial Biomarkers of High-Risk Exposure to EDCs in Pediatric Age

**Figure 3:**
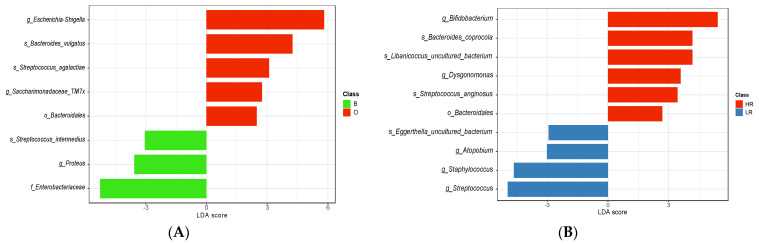
Based on the LefSe method, metataxonomic differences in fecal microbiota of infants: (panel (**A**)) breastfed by mothers showing urinary BPA concentrations over (O) or below (B) the BPA median value, (panel (**B**)) at high−risk (HR) and low−risk (LR) exposure to phthalates.

A Linear Discriminant Effect Size (LEfSe) analysis was conducted using all fecal samples (T_0_, T_1_, T_2_, and T_3_) collected during the first year of the infant’s life. Setting the threshold at an LDA-score > 2 and *p* < 0.05, eight different taxa reached significance in the comparison of O against B infants, while ten taxa were significantly different between HR and LR infants (Figure 3A and Figure 3B, respectively). Considering the entire first year of life, the *Bacteroidales* order, two genera (*Escherichia-Shigella*, *Saccharimonadaceae* TM7x), and two species (*Bacteroides vulgatus* and *Streptococcus agalactiae*) were significantly representative of infants belonging to the O-subset (Figure 3A). In contrast, *Enterobacteriaceae*, *Proteus*, and *Streptococcus intermedius* were the most representative taxa of infants belonging to the B-subset (Figure 3A). Evaluating differences in gut microbiota between HR and LR infants, the order *Bacteroidales*, the genera *Bifidobacterium* and *Dysgonomonas*, and the species *Bacteroides coprocola*, *Libanicoccus* uncultured bacterium, and *Streptococcus anginosus* dominated in HR (Figure 3B). The opposite condition (i.e., LR) was instead supported by the presence of three different genera (*Atopobium*, *Staphylococcus*, and *Streptococcus*) and one uncultured species of *Eggerthella* (Figure 3B).

### 3.6. Over/Under-Expressed Metabolic Pathways in Pediatric Gut Microbiota under High-Risk Exposure to EDCs

**Figure 4:**
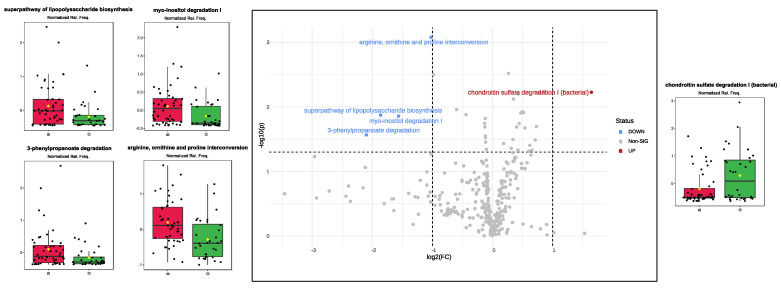
Volcano plot illustrating the upregulated (in red) and downregulated (in blue) KEGG pathway relative frequencies in infants exposed to elevated levels of BPA. Boxplots display normalized values of KEGG pathway relative frequencies, with B indicating B−group infants and O representing O−group infants. Significance was achieved for pathways exhibiting a fold change (FC) > 2 (log2) and a *p*-value < 0.05 (expressed as –log_10_).

Using the PICRUSt2 software 2.0 version, predicted metabolic pathways were generated from 16S rRNA marker gene data, and their relative frequencies were compared as previously conducted between groups. When comparing B vs. O infants (Figure 4), four pathways (arginine, ornithine, and proline interconversion; super pathway of lipopolysaccharide biosynthesis; Myo-inositol degradation I; and 3-phenylpropanoate degradation) were significantly higher in the former group, whereas one pathway (bacterial chondroitin sulfate degradation I) significantly represented the latter group. Additional pathways reached the significance threshold; however, due to a small fold change (FC), these were not considered significant differences between B- and O-infants.

**Figure 5:**
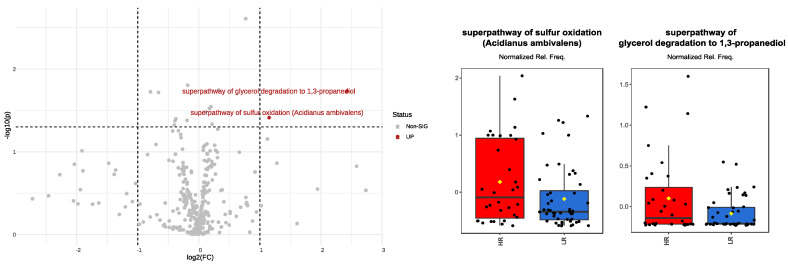
Volcano plot illustrating upregulated (in red) KEGG pathway relative frequencies in infants exposed to a high risk (HR) of endocrine-disrupting chemicals (EDCs). Boxplots, with red denoting HR–infants and blue representing LR–infants, display normalized values of KEGG pathway relative frequencies. Significance was attained for pathways exhibiting both a fold change (FC) > 2 (expressed as log2) and a *p*-value < 0.05 (expressed as –log10).

When the comparison accounted for infant stratification into HR vs. LR, only two pathways were found to be significantly different (the super pathway of sulfur oxidation (*Acidianus ambivalens*) and the super pathway of glycerol degradation to 1,3-propanediol), and both were overexpressed in HR infants (Figure 5). Additional pathways reached the significance threshold (*p* < 0.05); however, showing an FC of less than two, these were not considered as significant differences between HR and LR infants.

## 4. Discussion

The National Institute of Environmental Health Science (NIH) defines EDCs as exogenous factors interfering with endogenous hormones, leading to an increased risk of adverse outcomes on health [28]. Similarly, the polymicrobial community inhabiting the colonic milieu has demonstrated its role in the modulation of hormonal secretion [29]. Therefore, the current literature assigns to both gut microbiota and EDCs a pivotal role in the onset of the broad spectrum of NCDs, including type 2 diabetes (T2DM) and cancer, as well as obesity [30]. The knowledge of how EDC exposure in adulthood can cause adverse consequences on health is widely recognized, but early life exposures are demonstrated to have more severe effects persisting through life [31,32]. From birth, the gut microbiota engages in a bidirectional relationship with the host, and this interaction is influenced by various factors, including genetics, delivery mode, household exposures, type of milk feeding, and timing of weaning [33,34]. However, how gut microbiota development is shaped by different EDC exposure levels is still poorly investigated. A lack of evidence exists for EDC susceptibility during “the first 1000 days” of life, a timeframe in which sources of exposure mainly depend on the household and milk-feeding.

We indicate here the influence of maternal EDC levels and the effect exerted by these biomarkers on the gut microbiota of the baby. 

Specifically, we herein considered the entero-mammary circle as the process to transfer chemicals from the maternal bloodstream—via breast milk—to infants. With this purpose, we collected maternal urine samples, known as one of the most important biological fluids representative of the exposure level to EDCs, and screened them for EDC concentrations, retrospectively profiling the gut microbiota composition in infants who were breastfed up to 1 year of age. Concerning the EDC–gut microbiota axis, most of the available literature was provided by studies on animal models [35,36,37,38] and, on average, these works deeply focused on a few specific EDCs. For our analyses, instead, we concomitantly considered five different chemicals, among which the BPA was also analyzed, since it appears the most studied among EDCs [39]. Herein, BPA positively correlated with *Bacteroidia* at different taxonomic levels, i.e., from order to species, with *B. vulgatus* emerging as the most representative taxon of this relationship. BPA [40] and *Bacteroides* [37] shared common features according to evidence on how, although supported by different experimental studies, both demonstrated an obesogenic role. As far as it concerns *B. vulgatus*, it has also been suggested as a biomarker in T2DM patients [41]. Besides metabolic-related diseases, the potential carcinogenic contribution of BPA is a field of ongoing research [42]; however, nowadays, the literature does not completely agree on its correlation with tumoral diagnosis, due to the presence of many limitations that increase the risk of biased results [43]. Considerable attention has been paid to *Bacteroides* spp., particularly to the *B. vulgatus* and *B. fragilis* groups, which were found to be the main cancer-associated biomarkers according to large populational studies [44,45]. Based on these considerations, it seems possible to speculate about a possible boost effect provided by some specific gut microbiota taxa in exacerbating the risk of obesity, metabolic disease, and cancer onset when associated with high BPA exposure.

Based on our cohort stratification, the comparison between infants with low BPA exposure and those with high BPA exposure led us to examine how the *Ruminococcus torques* group displayed a noteworthy increase in infants whose mothers reported urinary BPA concentrations below the median values. In a prior investigation that explored small intestinal paracellular permeability and immune system development in healthy children during their first 2 years of life, it was observed that the *Ruminococcus torques* group was significantly linked to changes in calprotectin levels [46]. Furthermore, considering the role of the *Ruminococcus torques* group in mucin degradation processes [47], the authors concluded by suggesting a potential contribution of the *Ruminococcus torques* group to the maturation of the immune system during early life [46].

Conversely, we observed a contrasting trend for *Erysipelatoclostridium* and *Bifidobacterium* breve abundances within our cohort. These two taxa were found to be more prevalent in the gut microbiota of infants exposed to high BPA concentrations. Notably, members of the *Erysipelatoclostridium* genus exhibited a heightened immunogenic potential and demonstrated resistance against a wide range of antibiotics [48]. Additionally, a recent study by Chen et al. [49] discussed a potential association between *Erysipelatoclostridium* and both obesity and increased intestinal permeability in mouse models. This finding aligns with previous research [50] that, upon reviewing the literature, attributed a pivotal role to *Erysipelatoclostridium* in a broad spectrum of inflammatory processes. No concrete evidence is currently available to elucidate how both BPA and EDCs may influence the abundance of *Bifidobacterium*. *Bifidobacterium* species are widely recognized as crucial contributors to infant health, particularly during the first 6 months of life when exclusive breastfeeding is prevalent, as they play a significant role in immune system development [51,52]. While *Bifidobacterium* species have acted as probiotics, demonstrating the ability to reduce fasting blood glucose levels and alleviate insulin resistance across various study populations [53], it is worth noting that prior research on type 2 diabetes (T2D) has identified an increase in *Bifidobacterium* taxa within the gut microbiota of patients [54,55].

In substitution of Firmicutes presence in the gut lumen, *Bifidobacterium* can be involved in metabolic processes, allowing the extraction of energy from diet [56]. In this context, an investigation targeting gut microbiota composition in a Pakistani T2D cohort revealed a positive correlation between *Bifidobacterium* abundance and *Libanicoccus* spp., both of which were increased in individuals with the disease [54]. Consistent with this finding, the LEfSe-based analysis conducted in this study revealed a similar enrichment of both *Bifidobacterium* and *Libanicoccus* in infants at high risk (HR). Recent developments in metabolic-associated diseases have also indicated that *Libanicoccus* promoted atherosclerosis in vivo [57,58].

In contrast, Proteobacteria yielded controversial findings. Elevated BPA exposure led to the highest LEfSe score for the Escherichia-Shigella genus, while the *Enterobacteriaceae* family, driven by the contribution of the *Proteus* genus, exhibited the opposite trend. Although changes in Proteobacteria and, consequently, proteolytic metabolic processes, are well-established in the context of gut imbalances associated with adverse outcomes stemming from the presence of opportunistic pathogens within this phylum [59,60], the analysis of blood, urine, or stool samples in a prior study [61] did not reveal any direct correlation between Proteobacteria abundance and BPA concentration.

The gut microbiota was also investigated as a unique machine providing accessory enzymes to hosts, leading to health or disease. Performing a PICRUSt2 analysis, we observed increased expression of arginine, ornithine, and proline interconversion in infants with lower exposure to BPA levels. Amino acids, including arginine, ornithine, and proline, orchestrate essential physiological processes, ensuring cellular balance, and these were found to be under-regulated in infants exposed to high levels of BPA. Amino acids, indeed, are pivotal in protein synthesis, the urea cycle, and collagen formation. The tightly regulated interconversion of these amino acids is essential for maintaining normal cellular function [62]. Disruptions in this balance can lead to urea cycle disorders, rare genetic conditions impairing ammonia detoxification and causing its toxic accumulation in the blood, which is particularly harmful to the brain [63,64]. As an additional pathway poorly featured in O-infants, 3-Phenylpropanoate, or hydrocinnamate, is a compound that is usually detectable in foods. Its unremarkable metabolism, under normal circumstances, contributes to overall energy metabolism, allowing us to speculate about the possibility that O-infants might be exposed to an increased risk of obesity and metabolic disease later in life [65]. This field of discussion was further supported by results concerning myo-inositol, a polyol that plays diverse roles in cellular processes, potentially benefiting gut health and insulin sensitivity [66,67], and was, once again, found to be under-expressed in O-infants. All this evidence emphasizes the intricate relationship between microbes and metabolic well-being and, as the focus of the present study, our results were obtained by an infant stratification considering a high to low maternal exposure at different levels of EDCs, therefore opening new questions about the possibility that high maternal EDC exposure can lead the infant, via breastfeeding, to experience adverse health issues later in life.

## 5. Conclusions

Although our results partially reproduce those provided by previous studies, they cannot be considered conclusive due to different limitations. One of the most important limitations is the lack of concrete EDC quantification in infants’ biological fluids. Furthermore, the limited sample size makes this research a pilot study that requires further investigation in a larger population. Regarding the existence of host genetic inherited traits, considering this limitation, the present study sheds light on the involvement of some microbiota, whose presence, abundance, and genomic functions actively lead to an increase in the risk of future onset of NCDs. *Bacteroidia*, particularly the *B. vulgatus* group, reported various evidence about its possible positive relationship with high BPA and phthalate exposure levels. The concomitant increase in *Bifidobacterium* and *Libanicoccus* abundance in gut microbiota needs further study on the possible role in acting as biomarkers of high phthalate exposure levels. However, considering the absence of great evidence concerning EDC exposure in the first years of life, various questions about the ECD–gut microbiota axis are still open to answer, and further research is needed to improve the knowledge of the mechanisms that may be involved in NCD onset in adulthood.

## Figures and Tables

**Table 1:** Maternal urinary concentrations of EDCs. Bisphenol A (BPA) and phthalate levels (µg/g creatinine) are reported as median, smallest to biggest value (min-max), and interquartile range (IQR, 25th–75th percentile).

	BPA	MEP *	MIbP *	MEHHP *	MBzP *
median	0.96	7.73	0.89	1.24	0.64
min–max	0.34–2.98	0.01–83.67	0–10.4	0.1–10.28	0.16–6.77
IQR	0.73–1.63	3.05–12.84	0–2.99	0.79–2.23	0.42–1.03

* Mono-ethyl phthalate (MEP); mono-isobutyl phthalate (MIbP); mono-(2-ethyl-5-hydroxylhexyl) phthalate (MEHHP), and mono-benzyl phthalate (MBzP).

**Table 2:** Based on the Principal Coordinate Analysis (PCoA), β-diversity (Jaccard’s index) differences (PERMANOVA) between infants’ fecal samples delivered at different timepoints (meconium—T_0_; 1 month of age—T_1_; 6 months of age—T_2_; and 12 months of age—T_3_). Comparisons considered infant stratification in those who were exposed to high (O) or low (B) BPA concentrations via breastfeeding, or those who were at high-risk (HR) or low-risk (LR) exposure to other EDCs via breastfeeding.

	O |vs.| B			HR |vs.| L		
Taxonomic Level	F-Value	R-Squared	*p*-Value	F-Value	R-Squared	*p*-Value
Phylum	1.687	0.021	0.150	1.574	0.019	0.161
Class	1.191	0.015	0.299	1.662	0.020	0.113
Order	1.036	0.013	0.405	1.418	0.017	0.144
Family	1.133	0.014	0.319	1.539	0.019	0.105
Genus	1.716	0.021	0.049 *	1.483	0.018	0.090
Species	1.263	0.015	0.239	1.360	0.017	0.178

* Statistically significant *p*-value.

## Data Availability

All data presented in this study, not yet publicly archived, shall be made available by the corresponding author on request.

## Supplementary Materials

The following supporting information can be downloaded at: https://www.mdpi.com/article/10.3390/biomedicines12010234/s1. Table S1. General characteristics of mothers included in the study (n = 20); Table S2. Maternal lifestyle habits [68,69]; Table S3. Phthalate and BPA exposure determinants [70]; Figure S1. Timeline of sample collection of the A.MA.MI (*Alimentazione Mamma e bambino nei primi MIlle giorni*) project.
