# Supplementary material for: Maternal Exposure to Endocrine-Disrupting Chemicals: Analysis of Their Impact on Infant Gut Microbiota Composition
Source: Biomedicines. 2024 Jan 19;12(1):234. doi: 10.3390/biomedicines12010234 (PMC10813257; doi:10.3390/biomedicines12010234)
Supplement: Supplementary file 1 [file biomedicines-12-00234-s001.zip › biomedicines-2803717-supplementary.pdf]

Supplementary material

Table S1. General characteristics of mothers included in the study (n = 20). Data were reported as mean ± standard deviation (SD) or median and interquartile range [Q1–Q3] or frequency of subjects expressed as a percentage (%).

Variables (Cutoff Value or Reference Interval)	Mean ± SD; Median [Q1–Q3]; %
Age (years)	34
BMI (kg/m2)	
Underweight (BMI < 18.5)	
Normal weight (18.5 ≤ BMI < 25)	23.6 ± 4.9
Overweight (25 ≤ BMI < 30)	
Obesity (BMI ≥ 30)	
WC (<88 cm)	81,7 ± 13.4
WHtR (<0.5)	0.5 ± 0.08

Legend. BMI, Body Mass Index; WC, Waist Circumference; WHtR, Waist to Height Ratio

Table S2. Maternal lifestyle habits. Data were reported as mean +/- standard deviation, median, and interquartile range [Q1-Q3] or frequency of subjects expressed as number (n)and percentage (%).

Lifestyle Variables	Mean +/- SD; Median [Q1-Q3]; %
FF section—food frequency (score)	39.3 ± 4.3
Less healthy, n (%)	7 (35)
Moderately healthy, n (%)	7 (35)
Healthiest, n (%)	6 (30)
FH section—food habits (score)	34.3 ± 4.8
Inadequate eating habits, n (%)	7 (35)
Partially satisfactory eating habits, n (%)	7 (35)
Satisfactory eating habits, n (%)	6 (30)
Physical activity level—IPAQ (METs)	1386 [693-2772]
Sedentary (total METs <699, MET-min), n (%)	8 (40)
Moderate (total METs: 700–2519, MET-min), n (%)	5 (25)
High (total METs >2520, MET-min), n (%)	7 (35)
Smoking habit	
Smokers (%)	15%
Non-smokers (%)	85%

Legend. FF, food frequency section [68]; FH, food habits section [68]; IPAQ, International Physical Activity Questionnaire [69]; MET, metabolic equivalent of task.

Table S3. Phthalate and BPA exposure determinants. Phthalate and BPA exposure determinants were explored through a structured interview extrapolating questions from the LIFE PERSUADED ("Phthalates and bisphenol A biomonitoring in Italian mother–child pairs: link between exposure and juvenile diseases") Project questionnaire [70]. Data are listed as the frequency of subjects that report lifestyle habits. The determinants of exposure are expressed as percentages.

Determinants of exposure	Percentage
Use of plastic products for daily personal care (lotions, make-up, dental care, glasses, gloves)	75%
Presence of PVC in home, working and recreational environments	50%
Consumption of packaged food in plastic containers or film-wrapped food, canned (or tetra packed) food	75%
Consumption of packaged food or pre-cooked food to heat	20%
Cooked takeaway food consumption	60%
Disposable plastic use	10%
Frequent use of plastic containers for storage of cooked or raw food	65%
Use of dishwasher to clean plastic containers	35%
Use of film wrap to package and store food for consumption	65%
Use of microwave to cook or defrost food	60%
Regular use of plastic utensils for cooking	40%
Consumption of water from plastic bottles	40%
Consumption of sauces or dressings in plastic containers	50%
Packaged dressings or condiments from the original packaging	70%

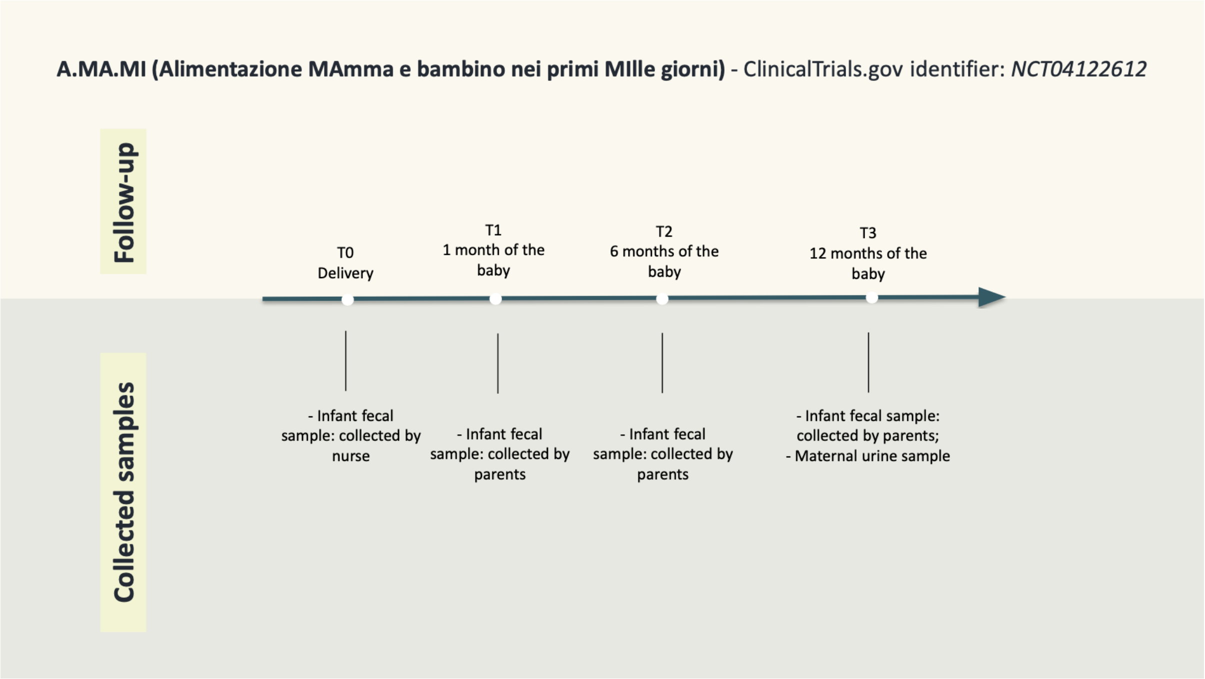

Figure S1. Timeline of sample collection of the A.MA.MI (Alimentazione Mamma e bambino nei primi MIlle giorni) project [13].
